# Characteristics and outcome of breast cancer-related microangiopathic haemolytic anaemia: a multicentre study

**DOI:** 10.1186/s13058-021-01386-y

**Published:** 2021-01-19

**Authors:** Marion Alhenc-Gelas, Luc Cabel, Frederique Berger, Suzette Delaloge, Jean-Sebastien Frenel, Christelle Levy, Nelly Firmin, Sylvain Ladoire, Isabelle Desmoulins, Pierre-Etienne Heudel, Florence Dalenc, Delphine Loirat, Coraline Dubot, Perrine Vuagnat, Elise Deluche, Meriem Mokdad-Adi, Anne Patsouris, Josselin Annic, Lounes Djerroudi, Marion Lavigne, Jean-Yves Pierga, Paul Coppo, Francois-Clement Bidard

**Affiliations:** 1 Department of Medical Oncology, Institut Curie, Paris and Saint Cloud, France; 2 UVSQ, Université Paris-Saclay, 35 rue Dailly, Saint Cloud, 92210 France; 3 Institut Curie, Biometry Unit, Paris and Saint-Cloud, France; 4 Department of Cancer Medicine, Institut Gustave Roussy, Villejuif, France; 5 Department of Medical Oncology, Institut de Cancérologie de l’Ouest, Saint-Herblain, France; 6 Department of Medical Oncology, Centre François Baclesse, Caen, France; 7 Department of Medical Oncology, Institut du Cancer de Montpellier, Institut de cancérologie de Montpellier INSERM U1194, Montpellier, France; 8 Department of Medical Oncology, Centre Georges-François Leclerc, Dijon, France; 9 Department of Medical Oncology, Centre Léon Bérard, Lyon, France; 10 Department of Medical Oncology, Institut Claudius Regaud, Institut Universitaire du Cancer de Toulouse-Oncopole (IUCT-Oncopole), Toulouse, France; 11 Department of Pathology, Institut Curie, Paris, France; 12 Université de Paris, Paris, France; 13 Reference Center for Thrombotic Microangiopathies (CNR-MAT), AP-HP.SU, INSERM UMRS, 1138 Paris, France; 14 Sorbonne University, Paris, France

**Keywords:** Microangiopathic haemolytic anaemia, Breast cancer, Survival, Prognostic factors

## Abstract

**Background:**

Cancer-related microangiopathic haemolytic anaemia (MAHA) is a rare but life-threatening paraneoplastic syndrome. Only single cases or small series have been reported to date. We set up a retrospective multicentre study focusing on breast cancer-related MAHA.

**Methods:**

Main inclusion criteria were known diagnosis of breast cancer, presence of schistocytes and either low haptoglobin or cytopenia and absence of any causes of MAHA other than breast cancer, including gemcitabine- or bevacizumab-based treatment. Patient characteristics, treatments and outcome were retrieved from digital medical records.

**Results:**

Individual data from 54 patients with breast cancer-related MAHA were obtained from 7 centres. Twenty-three (44%) patients had a breast tumour with lobular features, and most primary tumours were low grade (grade I/II, *N* = 39, 75%). ER+/HER2−, HER2+ and triple-negative phenotypes accounted for *N* = 33 (69%), *N* = 7 (15%) and *N* = 8 (17%) cases, respectively. All patients had stage IV cancer at the time of MAHA diagnosis. Median overall survival (OS) was 28 days (range 0–1035; Q1:10, Q3:186). Independent prognostic factors for early death (≤ 28 days) were PS > 2 (OR = 7.0 [1.6; 31.8]), elevated bilirubin (OR = 6.9 [1.1; 42.6]), haemoglobin < 8.0 g/dL (OR = 3.7 [0.9; 16.7]) and prothrombin time < 50% (OR = 9.1 [1.2; 50.0]). A score to predict early death displayed a sensitivity of 86% (95% CI [0.67; 0.96]), a specificity of 73% (95% CI [0.52; 0.88]) and an area under the curve of 0.90 (95% CI [0.83; 0.97]).

**Conclusions:**

Breast cancer-related MAHA appears to be a new feature of invasive lobular breast carcinoma. Prognostic factors and scores may guide clinical decision-making in this serious but not always fatal condition.

**Supplementary Information:**

The online version contains supplementary material available at 10.1186/s13058-021-01386-y.

## Background

Thrombotic microangiopathy (TMA) is a rare syndrome combining diffuse microvessel thrombosis and mechanical haemolytic anaemia, often associated with thrombocytopenia [1]. It can result in severe, life-threatening organ dysfunction, especially affecting the kidneys and central nervous system. TMA is a causally heterogeneous syndrome related to several conditions including thrombotic thrombocytopenic purpura (TTP) and haemolytic-uraemic syndrome (HUS), which are primarily caused by a functional deficiency of ADAMTS 13 (an enzyme involved in the degradation of Von Willebrand Factor) activity and Shiga toxin or complement dysregulation, respectively. TMA may also occur following exposure to certain drugs [1], including bevacizumab and gemcitabine, two antineoplastic agents that have been approved for metastatic breast cancer. TMA has also been observed in patients with solid tumours: cancer-related microangiopathic haemolytic anaemia (MAHA) is a rare paraneoplastic syndrome, first described as a clinicopathological entity in 1979 by Antman et al. [2]. Pathogenesis of cancer-related MAHA remains unknown; three mechanisms might be involved: (i) mechanical lysis of red blood cells, related to tumour micro-emboli in micro-vessels; (ii) inflammatory syndrome following activation of endothelial cells by circulating tumour cells; (iii) activation of the coagulation cascade (high tissue factor expression by endothelial and tumour cells; mucins secretion by tumour cells; von Willebrand Factor release caused by long-lasting bone marrow metastasis) [3–9]. Over the last 40 years, single cases or small retrospective series of cancer-related MAHA have been reported, with very poor survival [4–7]. Apart from lymphomas, most cases were reported in patients with adenocarcinoma, while very few cases have been reported with squamous cell carcinomas. In 2012, Lechner et al. performed a literature search and compiled 168 published cases of cancer-related MAHA; gastric and breast adenocarcinomas were the two most common primary tumour types in patients with cancer-related MAHA, accounting for 26% and 21% of compiled cases, respectively [10]. To date, despite a handful of case reports [5, 11–14], breast cancer-related MAHA remains a very poorly known condition. We therefore undertook a multicentre retrospective study to specifically identify breast cancer-related MAHA characteristics, outcomes and prognostic factors.

## Methods

This study was approved by the *Institut Curie* review board; a waiver of informed consent was granted because of the retrospective nature of the work.

### Eligibility criteria

The presence of schistocytes (> 0.5%) and either low haptoglobin or cytopenia (anaemia, thrombocytopenia or both) were mandatory for a diagnosis of MAHA, in agreement with current guidelines [15, 16]. Other eligibility criteria were patients with histologically proven breast cancer and MAHA diagnosed between 1995 and 2019 in participating centres. Ineligibility criteria were MAHA attributed to a cause other than breast cancer determined by treating physician and patients treated with either gemcitabine or bevacizumab at the time of or during the 6 months prior to MAHA diagnosis.

### Case search

In July 2018, a call for participants was sent to 13 French cancer centres, outlining the study’s objectives, and listing the data that needed to be collected for the study. Participating centres were encouraged, whenever possible, to automatically screen their patient files by means of computerised searches using the following key words (in French): “microangiopathie(s) thrombotique(s); micro-angiopathie(s) thrombotique(s); micro angiopathie(s) thrombotique(s); schistocyte(s)”. Computerised screening of laboratory registries was also performed, searching for blood counts with elevated schistocytes. Senior medical oncologists manually reviewed all cases retrieved by computerised search to confirm the diagnosis of breast cancer-related MAHA. In centres in which computerised screening was deemed unfeasible, physician-based case declaration was also accepted. Case collection was closed in February 2019.

### Statistics

Data requested from participating centres are listed in Additional file 1, Supp Mat 1A. The PRONOPALL score, a validated prognostic score in oncology patients [17], was obtained from collected data. Because of the rarity of breast cancer-related MAHA, no data apart from those required for the diagnosis of MAHA were considered to be mandatory. The call for participants indicated that the study would investigate patient characteristics, response to treatment, outcomes and prognostic factors. In the absence of robust breast cancer-related MAHA data in the literature, no hypothesis could be formulated concerning the number of cases needed to achieve any of the study’s objectives; we did not hierarchise objectives into primary or secondary and this exploratory study did not have a predefined power.

The patients’ clinical characteristics are expressed as numbers and proportions; the Chi-squared test or Fisher’s exact test was used to compare categorical variables. Median follow-up was estimated using the Kaplan-Meier method.

Overall survival (OS) was determined from the date of MAHA diagnosis until the date of death or last follow-up. Survival curves were established by the Kaplan-Meier method. Biological and clinical factors were tested using a log-rank test in univariate analysis. The survival time variable was binarised into 2 categories: “death before or at 28 days” versus “death after 28 days”. The Hmisc package was used for imputation of missing data using the “aregImpute” function and to perform univariate and multivariate logistic regressions using “fit.mult.impute” with “lrm” as modelling function [18, 19]. Factors considered useful according to clinical considerations or with *p* value less than 0.2 in univariate analysis were included in a stepwise top-down procedure using the Akaike information criterion (AIC) and the likelihood ratio test as a criterion for variable selection. The accuracy of the final model was verified by controlling calibration and discrimation with the RMS package. The model with the lower value of Brier Score and the higher value of R^2^ was selected as the final model to ensure the best discrimination. Calibration was assessed by visual examination of the calibration plot generated after bootstrap resampling. A prognostic score was constructed and weighted with β-coefficients estimation in the final model. The discriminatory capacity of the score, which represents the probability of dying within 28 days after the diagnosis of MAHA, was estimated by calculating the sensitivity and specificity of the score with their 95% confidence intervals (95% CI). A ROC curve was displayed and the area under the curve (AUC) was calculated using the ROCit package [20]. The 95% CI of the AUC was estimated by bootstrap.

All statistical analyses were performed with R software (version 3.6.2). This report was written in accordance with the REMARK guidelines.

## Results

### Patients and tumour characteristics

**Fig. 1:**
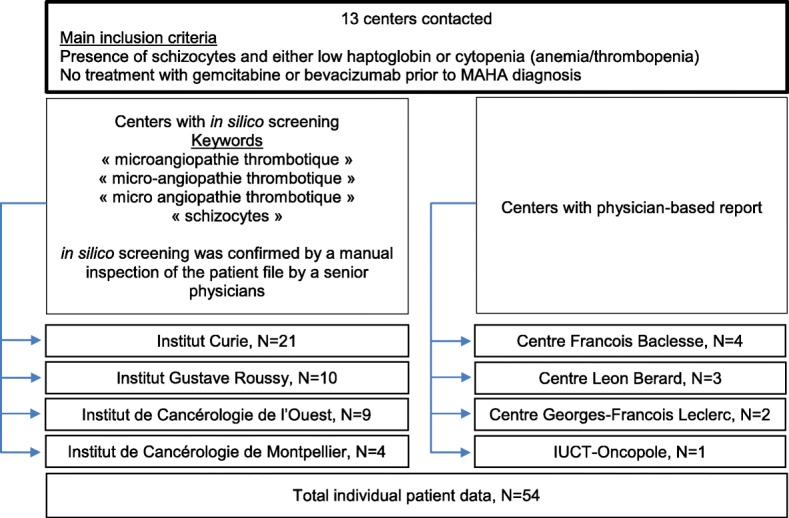
Study flow chart

Fifty-four cases of breast cancer-related MAHA from seven centres were included in this study. Forty-four cases were retrieved from four centres by means of computerised search, and ten cases were submitted by three centres based on the physician’s memory (Fig. 1). All patients were female. Median follow-up was 30.2 months (range 1.8–34).

Primary tumour characteristics, and clinical and laboratory features at MAHA diagnosis are shown in Tables 1 and 2, respectively. Invasive lobular adenocarcinoma or mixed adenocarcinoma with overt lobular component tumours was observed in *N* = 23 patients (44.2%). Few breast cancers were high-grade tumours (grade III, *N* = 13, 25.0%). Oestrogen receptor-positive/HER2-negative, HER2-positive, and triple-negative phenotypes accounted for *N* = 33 (68.7%), *N* = 7 (14.6%) and *N* = 8 (16.7%) breast cancers, respectively.

**Table 1:** Primary tumour characteristics (*N* = 54)

Characteristics	***N***	%
**Primary tumour size**		
T1–2	32	61.5
T3-T4	20	38.5
NA	2	
**Primary tumour histological type**		
IC-NST	29	55.8
Mixed with lobular component^a^ or ILC	23	44.2
NA	2	
**Nodal status**		
N0	14	26.9
N+	38	73.1
NA	2	
**Histological grade**		
Grade I–II	39	75.0
Grade III	13	25.0
NA	2	
**IHC profile**		
HR+/HER2−	33	68.7
HER2+	7	14.6
HR−/HER2−	8	16.7
NA^b^	6	

*NA* not available, *IHC* immunohistochemistry, *HR* hormone receptors, *IC-NST* invasive carcinoma of no special type, *ILC* invasive lobular carcinoma

^a^In mixed ductal–lobular carcinoma, the lobular component constitutes ≥ 50% of the tumour

^b^These 6 cases were diagnosed before 2000 and have missing HER2+ status

**Table 2:** Clinical and laboratory features at MAHA diagnosis and association with 4-week survival

		Univariate analysis				Multivariate analysis	
Factors	***N*** (%)	OR	95% IC	***p*** value	OR	95% IC	***p*** value
**PS at MAHA diagnosis**							
PS 1–2	30 (55.6%)	1.0		*0.003*	1.0		*0.01*
PS 3–4	24 (44.4%)	6.0	[1.8; 19.8]		7.0	[1.6; 31.8]	
**Clinical bleeding**							
Yes	9 (16.7%)	NS			NS		
No	45 (83.3%)						
**Neurological symptoms**							
Yes	15 (28.3%)	NS			NS		
No	38 (71.7%)						
NA	1						
**Dyspnoea**							
Yes	11 (20.8%)	NS			NS		
No	42 (79.2%)						
NA	1						
**Metastatic sites**							
< 3	25 (46.3%)	NS			NS		
≥ 3	29 (53.7%)						
**Metastasis**							
Bone marrow^a^	11 (78.5%)						
Bone	44 (81.5%)						
Lung	8 (14.8%)	NS			NS		
Liver	35 (64.8%)						
Other^b^	24 (44.4%)						
**Number of prior treatment lines**							
≤ 1	26 (48.1%)	1.0		*0.06*	NS		
> 1	28 (51.9%)	2.9	[1.0; 8.7]				
**Platelets**							
< 50 G/L	29 (53.7%)	NS			NS		
≥ 50 G/L	25 (46.3%)						
**Haemoglobin**							
< 8 g/L	26 (48.1%)	4.0	[1.3; 12.5]	*0.02*	3.7	[0.9; 16.7]	*0.08*
≥ 8 g/L	28 (51.9%)	1.0			1.0		
**Schistocytes**							
> 5.0%	10 (25.0%)	NS			NS		
0.5–5.0%	30 (75.0%)						
NA	14						
**Erythroblastemia**							
Yes	40 (85.1%)	NS			NS		
No	7 (14.9%)						
NA	7						
**Myelemia**							
Yes	38 (90.5%)	NS			NS		
No	4 (9.5%)						
NA	12						
**Prothrombin time**							
< 50%	8 (16.0%)	4.5	[0.9; 25.0]	*0.07*	9.1	[1.2; 50.0]	*0.03*
≥ 50%	42 (84.0%)	1.0			1.0		
NA	4						
**Fibrinogen**							
≤ 2 g/L	15 (34.9)	NS			NS		
> 2 g/L	28 (65.1)						
NA	11						
**Glomerular filtration eate**							
> 60 mL/min	25 (65.8%)	NS			NS		
30–60 mL/min	9 (23.7%)	NS					
< 30 mL/min	4 (10.5%)	NS					
NA	16						
**Total bilirubin level**							
< 1.24 mg/dL	16 (30.8%)	1.0		*0.007*	1.0		
≥ 1.24 mg/dL	36 (69.2%)	7.3	[1.8; 30.6]		6.9	[1.1; 42.6]	*0.04*
NA	2						
**Pronopall score**^c^							
Short survival	12 (31.6%)	3.7	[0.8; 16.8]	*0.09*			
Intermediate/long survival	26 (68.4%)	1.0			NS		
NA	16						

*NA* not available, *OR* odds ratio, *NS* non-significant

^a^Only 14 patients had a bone marrow examination (myelogram or bone marrow biopsy) at the time of BC-MAHA diagnosis

^b^Others metastatic sites: cerebral, carcinomatous meningitis, node involvement

^c^The pronopall score for early death among oncology patients was calculated according to Barbot et al., J Clin Oncol 2008, missing data being imputed

At breast cancer-related MAHA diagnosis, median age was 57 years (range 33–91) and all patients displayed breast cancer metastases. The median interval between diagnosis of breast cancer metastases and onset of breast cancer-related MAHA was 16.7 months (range 0–143.6); Kaplan-Meier Survival curves corresponding to time from the first cancer diagnosis and from the first metastasis until the development of MAHA are presented in Additional file 2, Supp Mat 2. None of the patients received mitomycin-C, gemcitabine or bevacizumab at the time or during the 6 months prior to MAHA for metastatic breast cancer. In *N* = 15 patients (28.0%), breast cancer-related MAHA was diagnosed either simultaneously or within 2 months of the diagnosis of metastatic breast cancer. Twenty-nine patients (53.7%) had three or more metastatic sites. Metastatic sites were mainly the bone (*N* = 44, 81.5%) and liver (*N* = 35, 64.8%), followed by the bone marrow (*N* = 11, 78.5%) and lung (*N* = 8, 14.8%). At MAHA diagnosis, 30 patients (55.6%) had a performance status (PS) ≤ 2; 9 patients (16.7%) presented clinical features of bleeding (ecchymosis, purpura, epistaxis, haematoma, haematuria and/or brain haemorrhage); 11 (20.8%) experienced dyspnoea, and 15 patients (28.3%) presented neurological symptoms (confusion, headache, dizziness, gait disorders, aphasia and/or somnolence), unrelated to presence of brain metastasis (not shown).

All patients had thrombocytopenia and anaemia. For 14 patients, elevated schistocytes (mandatory inclusion criterium) was reported in medical files, but exact counts were not available. Among 40 patients with available schistocyte counts, six (15.0%), 24 (25.0%) and 10 (25.0%) patients had schistocyte counts of 0.5 to 0.9%, 1.0 to 4.9% and 5.0% or higher, respectively. The clinical characteristics and outcome of the six patients with the lowest schistocyte counts were not significantly different from those of the other patients (not shown). Erythroblastemia and myelemia were commonly observed in 40 (85.1%) and 38 (90.5%) patients, respectively. Coagulation disorders (prothrombin time < 50%, platelets < 50G/L, fibrinogen < 1 g/L) were observed in six patients (11%), suggesting possible disseminated intravascular coagulation (DIC), according to the ISTH-DIC score (at least a score of 5) [21]. Impaired renal function, defined as glomerular filtration rate < 60 ml/min, was observed in 13 patients (34.2%). Thirty-six (69.2%) patients had elevated total bilirubin (≥ 1.24 mg/dL); Elevated bilirubin was not associated with presence of liver metastasis (Khi 2 test, *p* = 0.18). Other laboratory parameters at breast cancer-related MAHA diagnosis are shown in Table 2. Clinicopathological characteristics were not significantly different between cases retrieved by in silico screening and those reported by physicians (not shown).

### Survival and prognostic factors

**Fig. 2:**
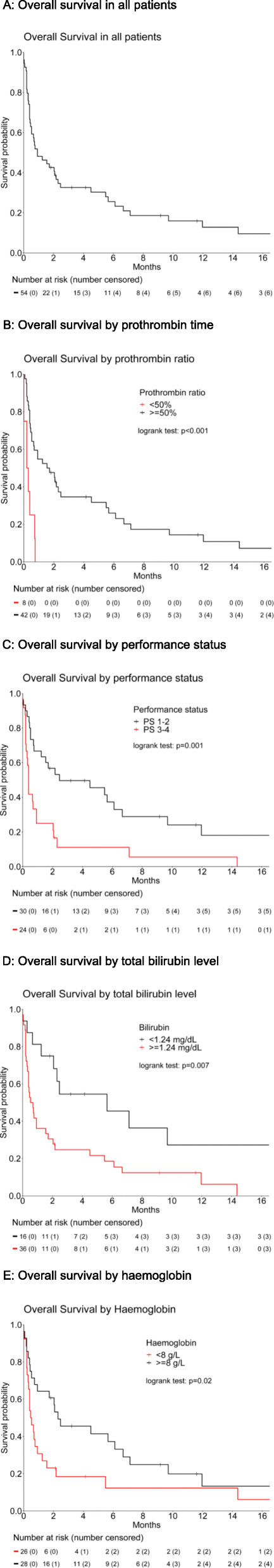
Overall survival according to independent prognostic factors. **a** Overall survival in all patients. **b** Overall survival by prothrombin time. **c** Overall survival by performance status. **d** Overall survival by total bilirubin level. **d** Overall survival by haemoglobin

**Fig. 3:**
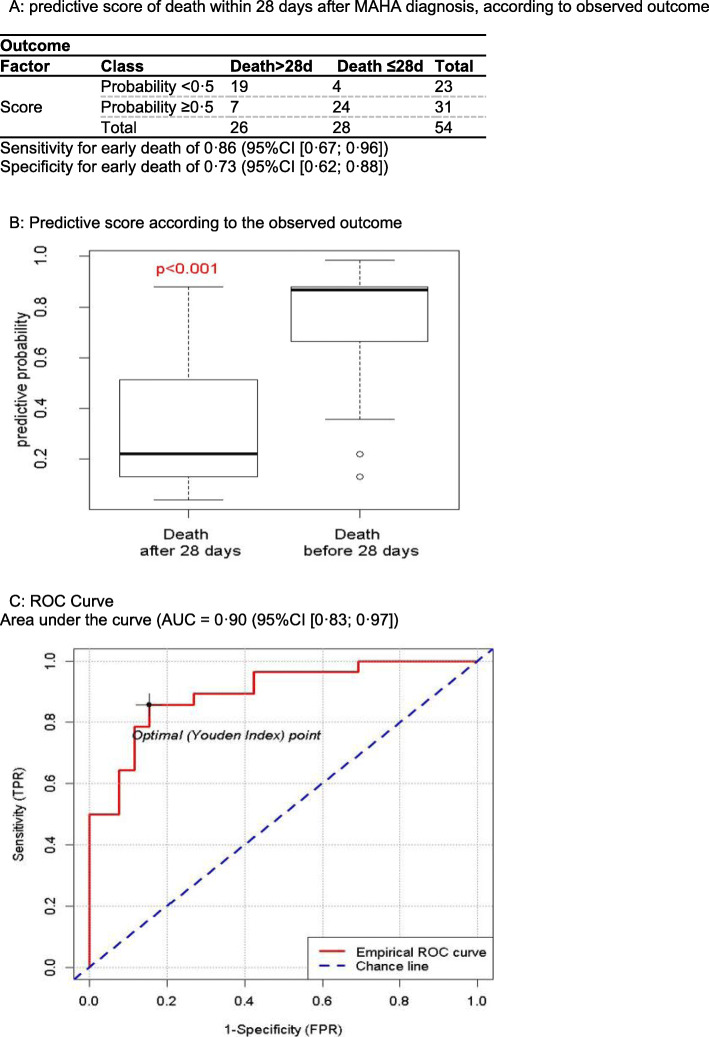
Breast cancer MAHA survival predictive score. **a** predictive score of death within 28 days after MAHA diagnosis, according to observed outcome. **b** Predictive score according to the observed outcome. **c** ROC curve. Area under the curve (AUC = 0.90 (95% CI [0.83; 0.97]). Sensitivity for early death of 0.86 (95% CI [0.67; 0.96]). Specificity for early death of 0.73 (95% CI [0.62; 0.88])

Median overall survival (OS) was 4.0 weeks (95% CI [2.3;10.7]); Fig. 2a).Three-, 6- and 12-month survival rates were 32.6% (95% CI [22.1; 48.1]), 25.6% (95% CI [15.9; 41.2]) and 12.8% (95% CI [5.7; 28.4]), respectively. The 23 patients who received no anti-tumour therapy after MAHA diagnosis had a median OS of 10 days (range 0–203; Q1: 5.5; Q3:18). Twenty-one patients received one line of anti-tumour therapy and achieved a median OS of 47 days (range 0–353; Q1: 15; Q3: 98). Ten patients received two or more successive lines of anti-tumour therapy and achieved a median OS of 290 days (range 52–1035; Q1: 149; Q3: 524). Only yen patients were still alive after 6 months, and only 5 were still alive at or after 1 year. Noteworthy, outcomes are dramatically poor compared to general metastatic breast cancer population without TMA [22]. To identify patients that could be eligible for palliative care, we then assessed factors associated with overall survival less than 4 weeks. In univariate logistic regression analysis, PS 3/4 (odds ratio (OR) = 6.0, 95% CI [1.8; 19.8]), one or more prior lines of treatment (OR = 2.9 [1.0; 8.7]), elevated bilirubin (OR = 7.3 [7.8; 30.6]), haemoglobin < 8 g/dL (OR = 4.0 [1.3; 12.5]), prothrombin time < 50% (OR = 4.5 [0.9; 25.0]) and a short survival according to the PRONOPALL score (after implementation of missing data) (OR = 3.7 [0.8; 16.8]) were associated with a higher risk of death within 4 weeks of MAHA diagnosis (Table 2). Fourteen clinical characteristics among the 24 available were included in the multivariate analysis based on their *p* value less than 0.20 or for clinical rationale (Additional file 1, Supp Mat 1B). In multivariate analysis, PS 3/4 (OR = 7.0 [1.6;31.8]), elevated bilirubin (OR = 6.9 [1.1;42.6]), haemoglobin < 8 g/dL (OR = 3.7 [0.9; 16.7]) and prothrombin time < 50% (OR = 9.1 [1.2; 50.0]) remained significantly associated with a higher risk of death within 4 weeks of MAHA diagnosis (Table 2). The corresponding survival curves are shown in Fig. 2b–e. The PRONOPALL score being not an independent prognostic factor for early death, we combined the independent prognostic factors into a breast cancer MAHA survival score (Fig. 3a). Applied to our retrospective cohort, this score displayed a sensitivity for early death (< 4 weeks OS) of 0.86 (95% CI [0.67; 0.96]), a specificity of 0.73 (95% CI [0.52; 0.88]) and an area under the curve (AUC) of 0.90 (95% CI [0.83; 0.97], Fig. 3).

## Discussion

To our knowledge, this is the first large cohort study of breast cancer-related MAHA addressing the clinical and laboratory characteristics at MAHA diagnosis and identifying survival prognostic factors. Fewer than 60 individual cases of breast cancer-related MAHA have been reported in the literature, mostly corresponding to single case reports [23]. The limited clinical and laboratory data available in these reports and a very likely publication bias (biased toward patients with exceptional survival) limit the value of case report compilations.

Firstly, our study confirms that breast cancer-related MAHA is very rare: we identified 54 cases over the last 20 years (1995–2018) in seven of the largest breast cancer centres in France. Patients with a schistocyte count higher than 0.5% were included in our study, while some guidelines recommend a 1.0% cut-off for the diagnosis of mechanical haemolysis [24, 25]. However, only six of our patients had a schistocyte count between 0.5 and 1.0%, and neither their clinical characteristics nor their outcome was significantly different from those of the other patients.

Our study did not estimate the breast cancer-related MAHA incidence rate, as the number of metastatic breast cancer patients treated over the same time period is unknown. However, for benchmarking purposes, in a single participating institution (*Institut Curie, Paris*), 2 patients were diagnosed with breast cancer-related MAHA and 91 patients were diagnosed with breast cancer meningeal carcinomatosis between 2000 and 2007 [26]. Meningeal carcinomatosis has an estimated cumulative incidence of < 5% in metastatic breast cancer patients [26, 27]. A cumulative incidence of ~ 0.1% is therefore likely for breast cancer-related MAHA among metastatic breast cancer patients. However, the short survival observed in our study suggests that many patients may die before MAHA is even diagnosed.

Secondly, regarding primary tumour characteristics, a new finding of our study is the high prevalence of breast adenocarcinoma with either lobular histology or overt lobular component (44.2%) compared to previous reports describing the metastatic breast cancer population (10–14%) [22, 28]*.* To our knowledge, this association has not been previously demonstrated: while four of the eight cases of breast cancer-related MAHA reported by Regierer et al. were lobular adenocarcinoma, the histological subtype was missing in the 36 cases compiled in the compilation of published cases by Lechner et al. [10, 14]. Interestingly, lobular breast adenocarcinoma and gastric adenocarcinoma, described as the leading cause of cancer-associated MAHA, share many phenotypic and genotypic traits in common, such as low E-Cadherin [29]. High mucin expression may also play a direct role in the pathogenesis of cancer-related MAHA, as it triggers platelet aggregation independently of tissue factor secretion [9, 30]. Regarding immunohistochemical profile, oestrogen receptor-positive/HER2-negative, HER2-positive and triple-negative subtypes frequency were similar to that observed in the general metastatic breast cancer population [22].

In accordance with previous MAHA reports, all patients had stage IV disease and many presented multiple metastatic sites [31–34] with laboratory signs of bone marrow involvement (myelemia, erythroblastemia) and/or cytologically-proven bone marrow metastasis [10, 33–37, 38]. Degradation fibrin markers (such as D-Dimers) were not available for most patients. However, coagulation disorders observed for 6 patients suggest possible DIC, according to ISTH-DIC criteria [21]. One of them presented with a low fibrinogen (< 1 g/L) and high D-Dimers, suggesting hyperfibrinolysis. To diagnose DIC in cancer, best strategy should be a longitudinal biological parameters monitoring including platelets, PT, fibrinogen and D-Dimers [39]. Unfortunately, due to the retrospective nature of our study, we were not able to perform it. Those are serious limitations for defining DIC in our cohort. Noteworthy, DIC can be responsible for biological disorders such as hemolysis, thrombocytopenia and schistocytes formation [40]. Then, it is almost impossible to know whether TMA is the origin or the consequence of coagulopathy. Establishing DIC frequency in a CR-MAHA population is challenging and, in practice, hard to determine [41].

Moreover, in keeping with prior reports focused on CR-MAHA [34], kidney and neurological disorders were rare, compare to other MAHA’s causes.

OS was very poor with a median OS of 4.0 weeks, shorter than that reported in some previous studies on cancer-associated MAHA [10, 34, 42]. Although a difference in survival specifically related to breast cancer-related MAHA cannot be ruled out, this difference compared to previous studies could be primarily attributed to our study method: most cases were retrieved by a systematic in silico search, while previous reports may be subject to declaration (to a MAHA registry [34]) or positive publication [10] biases.

To the best of our knowledge, no survival prognostic factors have yet been identified for breast cancer-related MAHA. In our study, altered performance status, abnormal prothrombin time, and elevated total bilirubin were the three strongest independent prognostic factors, while low haemoglobin had a more marginal impact. These factors could be used to distinguish patients likely to benefit from urgent antineoplastic therapy (the only effective treatment for CR-MAHA [34, 35, 38, 43–45]) from those who should preferably be referred for palliative care. Of note, the proposed algorithm was not validated on an external series, due to the rarity of breast cancer-related MAHA. Other limitations of our study include its retrospective nature, limited sample size and a lack of a systematic TMA diagnosis strategy including ADAMTS13 activity dosage to formally exclude idiopathic TTP. To prevent those bias, prospective studies should thus be performed to explore the incidence of CR-MAHA in metastatic breast cancer patients.

## Conclusions

Our study substantiates the pathological, clinical and laboratory profile of patients with breast cancer MAHA: patients with cancer of lobular or mixed-type histology associated with direct or indirect signs of bone marrow involvement and possibly DIC. We confirm the dramatically poor survival prognosis of breast cancer-related MAHA but identify prognostic factors that may be useful for treatment decision-making and any future clinical trial on breast cancer MAHA treatment.

## Supplementary Information

**Additional file 1: Supp Mat 1.** Clinical and biological parameters included in statistical analysis. 1A: Data requested from participating centres. 1B: Predictive score construction according to statistical analysis.

**Additional file 2: Supp Mat 2.** Kaplan-Meier survival curves corresponding to 2A: Time from first cancer diagnosis until development of MAHA. 2B: Time from first metastasis until development of MAHA.

## Data Availability

The datasets used and/or analysed during the current study are available from the corresponding author on reasonable request.

## Abbreviations

MAHA: Microangiopathic haemolytic anaemia
TMA: Thrombotic microangiopathy
PS: Performance status
AUC: Area under the curve
ROC: Receiver operating characteristic
OS: Overall survival
OR: Odds ratio
