# Supplementary material for: Characteristics and outcome of breast cancer-related microangiopathic haemolytic anaemia: a multicentre study
Source: Breast Cancer Res. 2021 Jan 19;23:9. doi: 10.1186/s13058-021-01386-y (PMC7814553; doi:10.1186/s13058-021-01386-y)
Supplement: Supplementary file 1 — Additional file 1: Supp Mat 1. Clinical and biological parameters included in statistical analysis. 1A: Data requested from participating centres. 1B: Predictive score construction according to statistical analysis. [file 13058_2021_1386_MOESM1_ESM.docx]

**Supp Mat 1. Clinical and biological parameters included in statistical analysis**

**1A: Data requested from participating centres**

| **Patient history** | Date of birth  Date of primary tumour diagnosis  Date of first metastasis  Date of MAHA diagnosis  Number of prior lines of treatment at MAHA diagnosis  Date of death or last follow-up |
| --- | --- |
| **Tumour characteristics** | Histological type  Tumour grade (I, II, III)  Hormone receptor (ER, PR)  HER2 status  Tumour size (T)  Node involvement (N)  Ki67 proliferative index (%)  Known BRCA1/2 mutational status  Metastatic sites at MAHA diagnosis |
| **Clinical and laboratory features**  **at MAHA diagnosis** | Performance Status (PS)  Clinical bleeding (yes, no, type of symptoms if any)  Dyspnoea (yes, no)  Neurological signs (yes, no, type of symptoms if any)  Haemoglobin (g/L)  Platelets (10^9^/L)  Leukocytes (10^9^/L)  Reticulocytes (10^9^/L)  Schizocytes (%)  Myelemia (yes, no)  Erythroblastemia (yes, no)  Prothrombin time (%)  Fibrinogen (g/L)  Albumine (g/L)  SGOT (ULN)  SPGT (ULN)  Total bilirubin (mg/dL)  Lactate dehydrogenase (ULN)  Haptoglobin (low or normal)  Glomerular filtration rate (ml/min) * |
| ER: Estrogen Receptor, PR: Progesterone Receptor  *Glomerular Filtration Rate was calculated using the Cockcroft-Gault formula  ULN: Upper Limit of Normal range | |

**1B: Predictive score construction according to statistical analysis**

| **Factors tested in univariate analysis** | **Factors tested in multivariate analysis^**^** | **Factors retained in the score** |
| --- | --- | --- |
| Albumine (g/L) |  |  |
| Clinical Bleeding |  |  |
| Dyspnoea |  |  |
| Erythroblastemia (yes, no) | Erythroblastemia (yes, no) |  |
| Fibrinogen (g/L) |  |  |
| Glomerular filtration rate (ml/min) ^*^ | Haemoglobin (g/L) | Haemoglobin (g/L) |
| Haemoglobin (g/L) |  |  |
| Haptoglobin (low or normal) |  |  |
| Lactate dehydrogenase (ULN) | Lactate dehydrogenase (ULN) |  |
| Leukocytes (109/L) |  |  |
| Metastatic sites |  |  |
| Myelemia (yes, no) |  |  |
| Neurological Symptoms |  |  |
| Number of prior treatment lines | Number of prior treatment lines |  |
| Number of metastatic sites | Number of metastatic sites |  |
| Performance Status (PS) | Performance Status (PS) | Performance Status (PS) |
| Platelets (G/L) | Platelets (G/L) |  |
| PRONOPALL score | PRONOPALL score |  |
| Prothrombin time (%) | Prothrombin time (%) | Prothrombin time (%) |
| Schistocytes (%) | Schistocytes (%) |  |
| SGOT (ULN) | SGOT (ULN) |  |
| SGPT (ULN) | SGPT (ULN) |  |
| Time between first metastasis diagnosis and diagnostic of MAHA syndrome | Time between first metastasis diagnosis and diagnostic of MAHA syndrome |  |
| Total bilirubin (mg/dL) | Total bilirubin (mg/dL) | Total bilirubin (mg/dL) |
| ^*^Glomerular Filtration Rate was calculated using the Cockcroft-Gault formula  ^**^14 clinical and biological characteristics among the 24 available were included in the multivariate analysis based on their p-value less than 0.20 in univariate analysis or for clinical rationale  ULN: Upper Limit of Normal range | | |
