# Supplementary material for: Characteristics and outcome of breast cancer-related microangiopathic haemolytic anaemia: a multicentre study
Source: Breast Cancer Res. 2021 Jan 19;23:9. doi: 10.1186/s13058-021-01386-y (PMC7814553; doi:10.1186/s13058-021-01386-y)

**Supp Mat 2. Kaplan-Meier survival curves corresponding to**

**2A: time from first cancer diagnosis until development of MAHA**

**
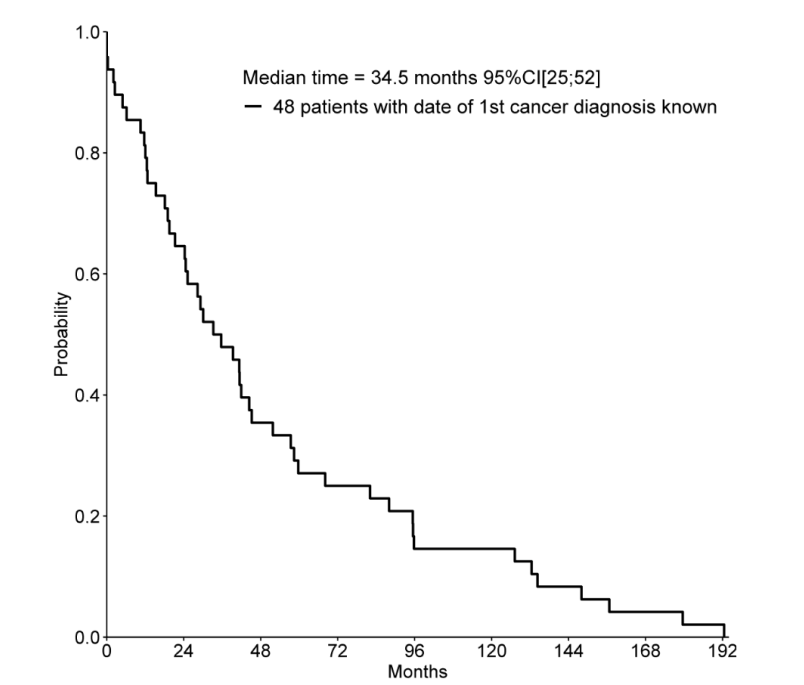
**

**2B: time from first metastasis until development of MAHA**


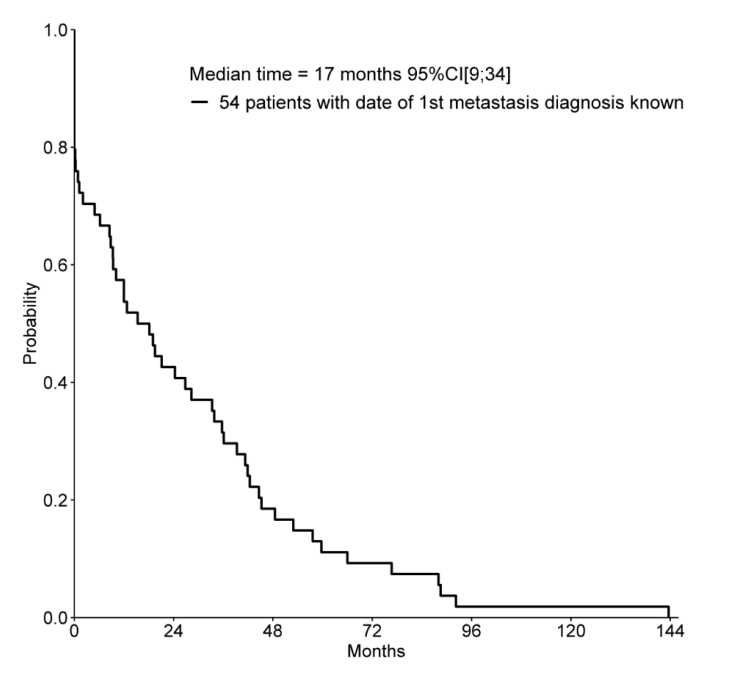

Supplement: Supplementary file 2 — Additional file 2: Supp Mat 2. Kaplan-Meier survival curves corresponding to 2A: Time from first cancer diagnosis until development of MAHA. 2B: Time from first metastasis until development of MAHA. [file 13058_2021_1386_MOESM2_ESM.docx]
